# Grade III Spleen Laceration After a Colonoscopy Treated with Splenic Artery Embolization: A Case Report

**DOI:** 10.7759/cureus.3843

**Published:** 2019-01-08

**Authors:** Mohamed Ahmed, Saba Habis, Rasha Saeed, Ahmed Mahmoud, Scott H Kwok

**Affiliations:** 1 Surgery, Riverside Community Hospital, Riverside, USA; 2 Internal Medicine, Riverside Community Hospital, Riverside, USA; 3 Radiology, Riverside Community Hospital, Riverside, USA

**Keywords:** colonoscopy, spleen laceration, coil embolization

## Abstract

Our patient is a 58-year-old female who presented to our emergency room with left upper quadrant abdominal pain the day after outpatient screening colonoscopy. A computed tomography (CT) scan of the abdomen and pelvis revealed a grade III spleen injury. She was admitted to our intensive care unit, and a gradual decline in her hematocrit was noticed. The patient did well and was discharged from hospital the day after splenic artery embolization.

## Introduction

Colonoscopy is the most common procedure for the diagnosis and treatment of lower gastrointestinal diseases, with fifteen million colonoscopies performed in the United States in 2012 [1]. A screening colonoscopy is a commonly performed outpatient procedure, with minimal morbidity. The reported complications rate averages 0.4%, which most commonly includes bleeding, perforation, and, rarely, splenic rupture [2]. There are just over 100 reported cases of splenic injury following colonoscopy in the literature, and it was first reported by Wherry et al. in 1974 [3]. The diagnosis is confirmed by a computed tomography (CT) scan of the abdomen and pelvis. We report a case of splenic rupture following colonoscopy, which was treated with splenic artery embolization.

## Case presentation

**Figure 1:**
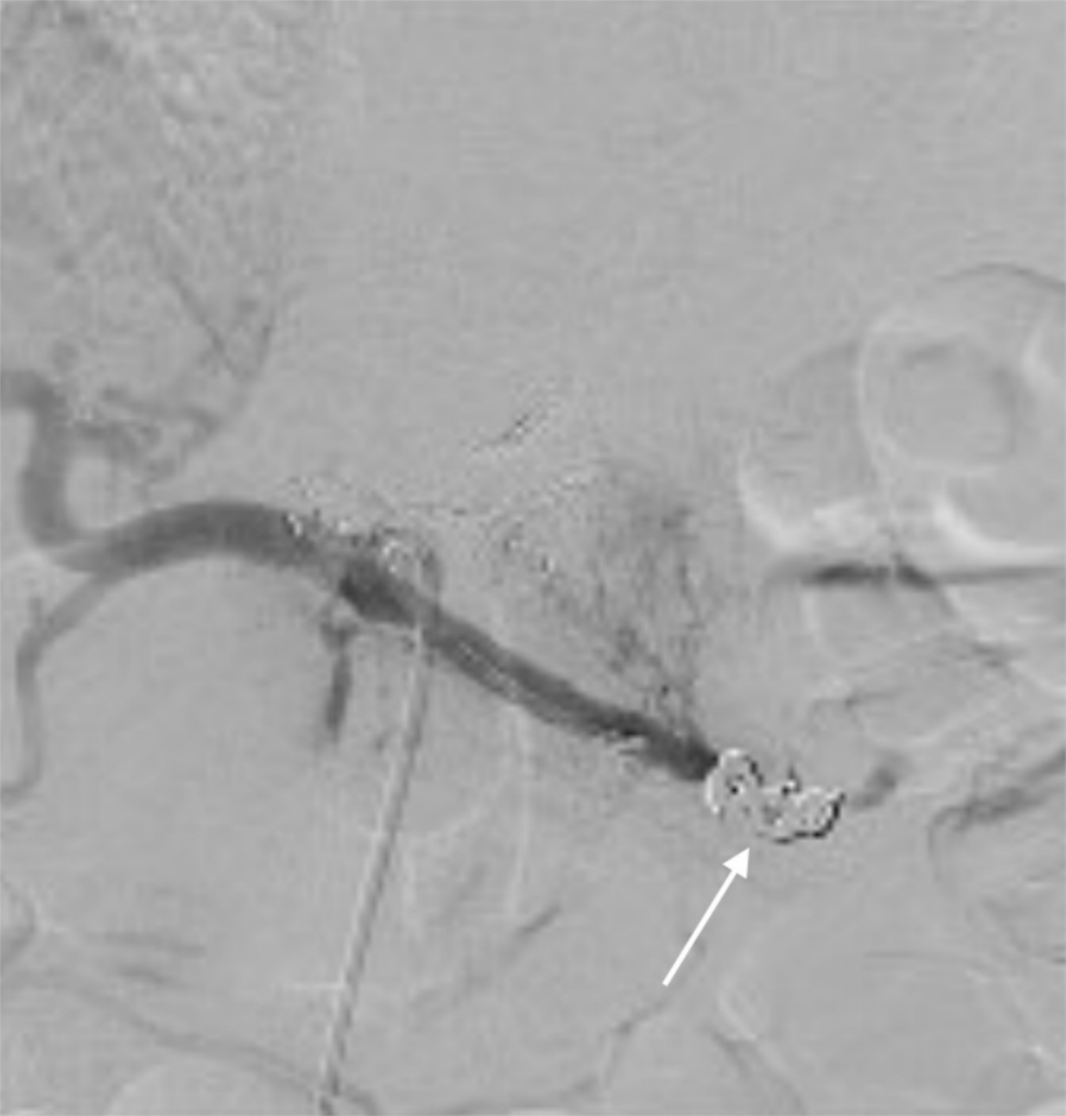
Angioembolization Splenic artery embolized, with no blood flow distal to the coils. Arrow indicates the interlocking coils

A 58-year-old female presented to our emergency room with worsening left upper quadrant abdominal pain radiating to her left shoulder the day after outpatient colonoscopy. A CT scan of the abdomen and pelvis revealed a grade III splenic injury with no obvious active extravasation (Figure 1).

**Figure 2:**
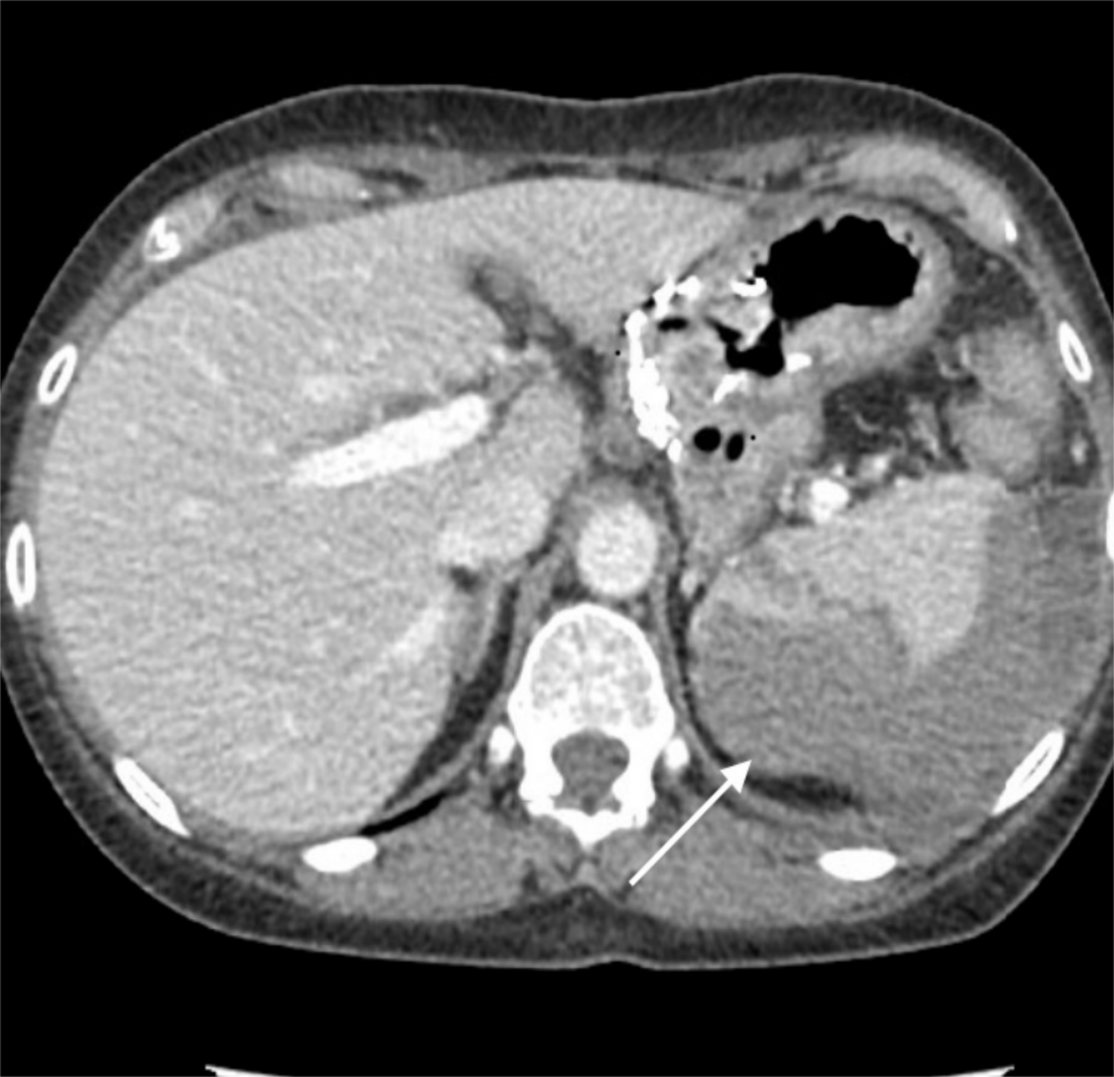
Grade III splenic injury with no obvious active extravasation

**Figure 3:**
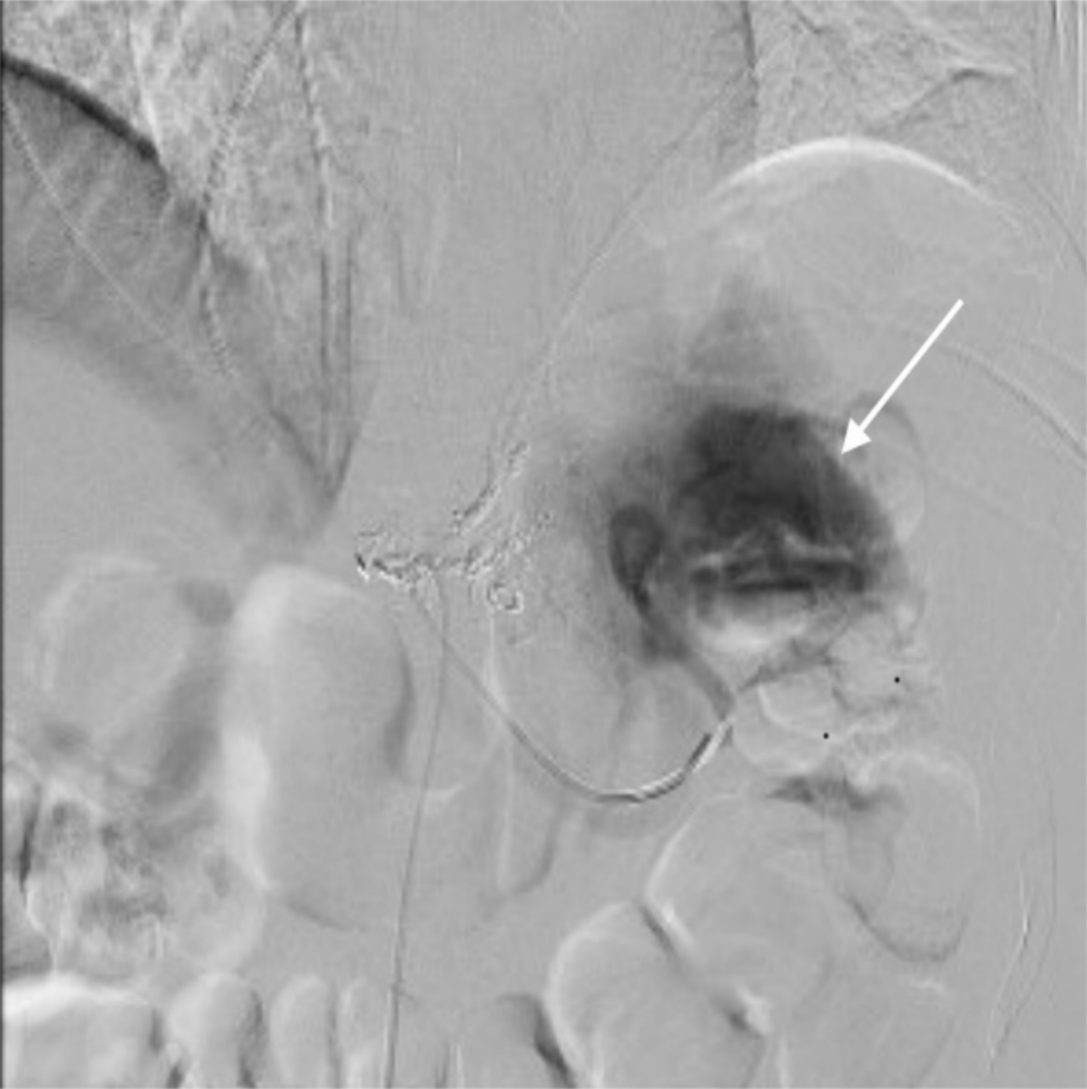
Splenic artery angiography Splenic artery angiography demonstrates active bleeding (blush), which is demonstrated by the white arrow

She was admitted to our intensive care unit and her initial hemoglobin (9 grams per deciliter) dropped gradually to 7.4 grams per deciliter over 24 hours. Angiography revealed a subcapsular blush (Figure 2). Splenic artery embolization, with interlocking coils, was performed (Figure 3). The patient did well with no further drop in her hemoglobin and was discharged from the hospital the following day.

## Discussion

The number of colonoscopies performed across the United States is increasing, and the potential complication of splenic injury should be on the examining physicians' differential [1]. Seventy-five percent of traumatic spleen injuries are managed conservatively, with good success, which does not appear to be the case in colonoscopy-induced injury. Available data suggest that 75% of these patients fail conservative management [4]. The majority of cases developed symptoms within 24 hours of the procedure, with abdominal pain being the most common presenting complaint (94%) and hypotension in 55% of the cases associated with a significant decrease in hematocrit. The CT scan of the abdomen and pelvis provides the most sensitive and specific method of diagnosis. Predictors of failed conservative management were grade II or above splenic laceration, old age, pre-existing splenic disease, hemodynamic instability, one unit of blood transfusion, hemoperitoneum, and female sex (75%) [5-6]. Neither a history of abdominal surgery nor the performance of a biopsy seems to increase the incidence of splenic injury [7]. In hemodynamically unstable patients, splenectomy is the definitive management. Nonoperative management was carried out successfully in 22% of patients who sustained a spleen injury after colonoscopy. Splenic artery embolization, which potentially preserves splenic function, was reported in a few cases [8].

## Conclusions

Splenic injury after colonoscopy is a rare complication and requires a high index of diagnostic suspicion when a patient presents with abdominal pain after colonoscopy associated with hemodynamic instability. While splenic injury after trauma can be managed conservatively in the majority of cases, this does not appear to be the case with a colonoscopy-induced injury. Angioembolization of the splenic artery appears to be very promising for the management of splenic ruptures.
